# Plasma cytokine levels and the presence of colorectal cancer

**DOI:** 10.1371/journal.pone.0213602

**Published:** 2019-03-18

**Authors:** Masaki Yamaguchi, Shin Okamura, Taiki Yamaji, Motoki Iwasaki, Shoichiro Tsugane, Vivek Shetty, Tomonobu Koizumi

**Affiliations:** 1 Shinshu University, Graduate School of Science & Technology, Department of Mechanical Engineering & Robotics, Ueda, Nagano, Japan; 2 Division of Epidemiology, Center for Public Health Sciences, National Cancer Center, Tokyo, Japan; 3 Center for Public Health Sciences, National Cancer Center, Tokyo, Japan; 4 Section of Oral & Maxillofacial Surgery, UCLA Health Sciences Center, Los Angeles, CA, United States of America; 5 Shinshu University School of Medicine, Department of Comprehensive Cancer Therapy, Matsumoto, Nagano, Japan; Katholieke Universiteit Leuven Rega Institute for Medical Research, BELGIUM

## Abstract

**Background/Aims:**

Cancer-related activation of cytokine networks are central aspects of tumor development. The goal of the study was to examine the possibility of plasma cytokines for the screening of colorectal cancer (CRC).

**Methods:**

We carried out a multicenter, hospital-based case-control study in 66 adult Japanese patients with CRC and 87 healthy adult Japanese. A multiplex bead array immunoassay was used to examine 27 different plasma cytokines. Their association with the presence of CRC was evaluated by logistic regression analysis after adjusting for potential confounding factors.

**Results:**

Thirteen plasma cytokines were notably associated with the presence of CRC (p< 0.05). Receiver operating characteristic analysis revealed that the combinatorial assessment of some of these plasma cytokines showed “good” capability for discriminating between CRC patients and control subjects (area under the curve (AUC): 0.819 for the combination of IL-9, Eotaxin, G-CSF, and TNF-α; 0.832 for the combination of IL-4, IL-8, Eotaxin, IP-10, and TNF-α). Individual cytokine assessments presented lower AUCs (0.657–0.755) than the combinatorial cytokine assessments.

**Conclusions:**

The levels of several plasma cytokines varied significantly between CRC patients and control subjects, suggesting the possibility of differentially expressed plasma cytokines as potential biomarkers for detecting the presence of CRC. Our results should be validated in other populations.

## Introduction

Colorectal cancer (CRC) is the second most common cancer occurring in females and the third most common cancer in males worldwide [1]. The disease affects 1.36 million people globally, accounting for nearly 10% of cancers [2]. CRC incidence and mortality continue to increase, owing mainly to population aging, and possibly to factors including a “westernized” diet, lifestyle, and lack of health-care infrastructure and resources.

Numerous studies have been carried out to optimize the early detection, diagnosis and treatment of this disease; CRC is one of the most-studied and best-characterized processes of tumorigenesis [3]. Current CRC screening methods can be generally grouped into invasive and non-invasive tests. The non-invasive stool-based tests currently available include the guaiac-based fecal occult blood test (gFOBT), the fecal immunochemical test (FIT), and the newer fecal DNA test [4]. These tests are based on the concept of detecting blood or shredded cell debris from vascularized polyps, adenomas and cancers. Owing to their simplicity and user-friendliness, gFOBT and FIT have found widespread use in CRC screening but are burdened by low sensitivity and specificity [5]. Invasive tests include flexible sigmoidoscopy and colonoscopy, have higher sensitivity and specificity because they offer direct visualization and pathology specimen collection [6]. However, routine use of such invasive tests for population-level screenings is not very practical given the increased risk of complications, higher associated costs, and limited capacity of the health-care system to perform such procedures. Furthermore, the logistics and discomfort involved with colonoscopies can cause at-risk patients to choose forgo screening entirely. Clearly, there is a need for alternates to gFOBT and FIT with higher sensitivity and specificity and are relatively inexpensive and straightforward to perform.

The ability to screen blood samples for tumor-related biochemicals has generated considerable interest in their use for detecting the presence of CRC early in its development. The hope is that the convenience of screening blood tests would allow for early diagnosis and treatment and lead to significant reductions in cancer-related morbidity and mortality [7]. Of the various pathways to tumor formation and progression, the inflammatory pathway has stimulated particular attention [8]. Several excellent reviews have described the cellular and molecular roles of inflammation in the development of cancer [9–13]. A central feature of activated immune cells is the production and release of growth factors and cytokines that modulate the inflammatory milieu in tumor tissues. Currently, more than 300 different cytokines have been identified. CRC has been linked to systemic and local changes in the cytokine profile [14], and recent work indicates that multiple pro-tumorigenic and also antitumorigenic cytokines are differently expressed in distinct CRC tissues [15]. Chemokines, small peptides that are structurally and functionally similar to growth factors, are also among the key players that promote cancer cell metastasis in some types of cancers. Chemokine ligand-receptor interactions have been reported to be involved in CRC progression [16]. Thus, the differential expression of the blood cytokines could have the potential in the early detection of CRC. To explore this, we carried out a multicenter, hospital-based case-control study to examine the associations between plasma cytokine levels and the presence of CRC. We hypothesized that the levels of some plasma cytokines would fluctuate depending on the presence of CRC and combinatorial cytokines would have greater discriminant ability.

## Material and methods

### Subjects

Details of the Nagano CRC study have been described elsewhere [17]. In brief, a multicenter, hospital-based case-control study was conducted at four hospitals in Nagano Prefecture, Japan (Matsushiro General Hospital, Saku General Hospital, Shinonoi General Hospital, and Hokushin General Hospital) between 1998 and 2002. Eligible cases were patients newly diagnosed with CRC during the study period at those hospitals. For each subject identified with CRC, the healthy controls (verified to be CRC-free) matched for age (± 3 years) and gender were randomly selected from the hospital’s health checkup program. All study subjects gave written informed consent for their participation in the study. Subjects completed a self-administered questionnaire concerning general and lifestyle characteristics (e.g. age, gender, height, weight, smoking, and drinking), as well as personal and family medical history, and provided blood samples before their treatment (CRC cases) or during their health checkup (healthy controls). After excluding nine ineligible cases (one mucinous carcinoma case, one squamous cell carcinoma case, and seven cases without an available blood sample) and their matched controls, 113 cases and 226 controls were included in the Nagano CRC study.

For the present study of plasma cytokines, we selected all of the 66 CRC cases (36 males and 30 females) aged 50–69 years with no current smoking and all of the 87 healthy controls (51 males and 36 females) within the same age range as cases and without current smoking, cancer, or surgery. Our study was approved by the Ethics Committee of Shinshu University School of Medicine (No.3361) and National Cancer Center (T2011-005) of Japan.

### Multi-analysis of cytokines

Collected blood samples were stored at –80°C and just prior to analysis thawed at 4°C in a refrigerator. Finally, the blood samples were brought to room temperature (24°C) before being added to the assay plate. After that, the blood samples were centrifuged at 1,500 × g for 15 min. A micropipette was then used to sample a fixed aliquot of each sample (50 μL) for subsequent analysis.

We used a multiplex bead array assay (Bio-Plex) that has previously been used to examine plasma cytokines [18,19]. Twenty seven cytokines (interleukin (IL)-1ra, IL-1β, IL-2, IL-4, IL-5, IL-6, IL-7, IL-8, IL-9, IL-10, IL12p70, IL-13, IL-15, IL-17A, C-C motif chemokine ligand 11 (CCL11; Eotaxin), fibroblast growth factor 2 (FGF-2), colony stimulating factor 3 (CSF3; G-CSF), colony stimulating factor 2 (CSF2; GM-CSF), interferon gamma (IFN-γ), tumor necrosis factor alpha (TNF-α), C-X-C motif chemokine ligand 10 (CXCL10; IP-10), C-C motif chemokine ligand 2 (CCL2; MCP-1), C-C motif chemokine ligand 3 (CCL3; MIP-1α), C-C motif chemokine ligand 4 (CCL4; MIP-1β), platelet-derived growth factor-BB (PDGF-BB), regulated on activation, normal T cell expressed and secreted (RANTES), and vascular endothelial growth factor (VEGF)) were analyzed according to the manufacturer’s instructions. The Bio-Plex multiplex bead array immunoassay system used human cytokine panels and the plates were read on a Bio-Plex Array Reader (Bio-Plex 200 System and Bio-Plex Manager Version 6.1, Bio-Rad Laboratories, Inc., Tokyo, Japan).

The results from the samples and their clinical information were entered into a structured database by research staff not directly involved in patient diagnosis, treatment, or sample examination. Patient diagnostic and pathological data were de-identified during sample collection and biomarker detection.

### Statistical analysis

All the statistical analyzes were performed with the Statistical Package for Social Sciences (SPSS) version 25 (Advanced Analytics, Inc. Tokyo, Japan). Unless otherwise stated, continuous data are summarized as the mean ± standard deviation (SD). A value of p < 0.05 was taken to represent statistical significance. The statistical analysis was not performed if the number of data points missing from one group exceeded half of all data points. In the statistical analysis, the plasma cytokine levels were subjected to log-transformation as well as standardizing, those less than the limit of detection were given a value of half the limit of detection [20, 21].

Prior to the construction of a prediction model, we compared mean plasma levels of each cytokine between cases and controls by using a multiple linear regression analysis with adjustment for gender, age, and hospital.

Next, a multiple logistic regression analysis was conducted to examine the association between plasma cytokines and the presence of CRC after adjustment for gender, age, and hospital. Odds ratios (OR) and their 95% confidence intervals (95% CI) for the presence of CRC were estimated for each of the plasma cytokines.

A correlation analysis was also performed for all of the pairwise of cytokines to evaluate their collinearity.

For the construction of a prediction model, candidate plasma cytokines were selected according to the following procedures. First, we nominated as the initial candidate the plasma cytokine that showed the smallest p-value in the aforementioned multiple logistic regression analysis, and excluded all cytokines whose correlation coefficients with the selected cytokine exceeded 0.7. Then, we identified another candidate cytokine with the next smallest p-value, and eliminated some of the remaining cytokines in the case where their correlation coefficients with the second selected cytokine were above 0.7. These procedures were repeated until candidate cytokines were finalized. By applying a backward elimination method to a logistic regression model with all possible candidates of plasma cytokines and fixed variables of gender, age, and hospital, we constructed a prediction model for the presence of CRC [22].

The model performance was assessed by a discrimination test using receiver operating characteristic (ROC) analysis [23]. ROC curves were depicted to investigate the discriminatory power of the plasma cytokine levels. The areas under the curves (AUC) were calculated to provide an overall summary of the detection accuracy of the plasma cytokine levels, and were empirically classified into three levels: poor when 0.50 ≤ AUC < 0.69, good when 0.70 ≤ AUC < 0.89, and excellent when 0.90 ≤ AUC < 1.

## Results

### Multi-analysis of plasma cytokines in the CRC patients and controls

Table 1 summarizes the background of the subjects and the number of samples taken. The mean ages of the CRC patients and controls were 61.4 ± 5.60 and 60.9 ± 6.11 years, respectively. The body mass indices (BMI) of the CRC patients and controls were 23.05 ± 3.20 and 23.72 ± 2.76 kg/m^2^, respectively. No significant differences were observed for gender, age, height, weight, BMI, smoking status, and past histories. The clinical stages of the CRC patients consisted of 9 stage 0, 20 stage I, 13 stage II, 19 stage III, and 5 stage IV.

**Table 1:** Summary of the background of the subjects and the number of samples taken.

Background	CRC patients	Controls	p-value
Sample number (*n*)	66	87	―
Male/Female	36/30	51/36	0.641*^1^
Age (years)	61.4 ± 5.6	60.9 ± 6.1	0.708*^2^
Height (cm)	157.7 ± 8.1	159.9 ± 8.5	0.136*^2^
Weight (kg)	57.4 ± 9.1	60.9 ± 10.2	0.061*^2^
BMI (kg/m^2^)	23.1 ± 3.2	23.7 ± 2.8	0.263*^2^
Smoking status			0.882*^1^
Current	0	0	
Former	22	30	
Never	44	57	
Drinking status			0.001*^1^
Current	30	56	
Former	8	0	
Never	28	31	
Past history of diabetes	5	6	0.872*^1^
Past history of colorectal polyp	19	12	0.022*^1^

*^1^ Pearson's chi-square (χ^2^) test was conducted.

*^2^ Mann-Whitney test was conducted.

Table 2 shows the results of multi-analysis of plasma cytokines in the CRC patients and controls. Of the target 27 cytokines, only 24 plasma cytokines could be analyzed in the CRC patients and controls, owing to a manifest lack of sensitivity of the Bio-Plex system to the remaining three (IL-2, IL-15, and MCP-1). The plasma cytokine levels of the CRC patients ranged between 0.19 (VEGF) and 5,794.57 (RANTES) pg/mL. By contrast, the cytokine levels in the control subjects ranged between 0.01 (IL-17A) and 7,825.46 (RANTES) pg/mL. A four-digit difference was observed in the absolute values of the cytokine levels.

**Table 2:** Results of multi-analysis of cytokines; comparisons between CRC patients and controls by multiple linear regression analysis.

Cytokine	Serum (pg/mL)				p-value*^1^	N of LOD*^2^ CRC/Control
	Control		CRC			
	Mean ± SD	Max—Min Min	Mean ± SD	Max—Min Min		
IL-1ra	57.80 ± 58.37	477.25–3.88	56.28 ± 28.90	165.95–10.78	0.767	0/0
IL-1β	1.80 ± 1.92	14.40–0.04	1.66 ± 0.84	3.94–0.51	0.571	0/0
IL-2	―	―	―	―	―	―
IL-4	1.49 ± 0.67	3.31–0.23	2.09 ± 0.67	3.67–0.88	< 0.001*^3^	0/0
IL-5	8.86 ± 6.26	33.42–0.68	9.67 ± 5.66	29.07–1.76	0.201	0/0
IL-6	4.98 ± 8.03	39.30–0.11	4.69 ± 7.85	46.64–0.31	0.622	2/0
IL-7	9.78 ± 9.91	80.81–0.20	10.69 ± 6.97	28.29–0.37	0.398	0/0
IL-8	7.30 ± 3.03	20.05–2.38	9.78 ± 5.15	32.86–0.61	0.007*^3^	0/0
IL-9	20.60 ± 11.97	63.08–2.44	32.37 ± 17.03	113.69–10.00	< 0.001*^3^	0/0
IL-10	7.69 ± 12.82	81.98–0.08	6.57 ± 5.24	25.46–0.20	0.555	2/1
IL-12p70	8.40 ± 10.91	83.62–0.12	9.00 ± 6.87	36.60–0.39	0.213	0/0
IL-13	21.08 ± 22.09	144.17–0.54	21.01 ± 19.65	96.01–1.54	0.779	0/0
IL-15	―	―	―	―	―	―
IL-17A	36.43 ± 32.34	137.78–0.01	56.89 ± 45.41	197.97–0.73	0.009*^3^	0/2
Eotaxin	35.90 ± 16.99	145.14–8.90	43.61 ± 17.19	139.12–21.34	0.001*^3^	0/0
FGF-2	33.18 ± 50.44	469.35–1.47	30.06 ± 10.31	56.93–9.97	0.302	0/1
G-CSF	28.94 ± 15.51	75.48–2.25	39.38 ± 14.48	80.45–9.24	< 0.001*^3^	0/0
GM-CSF	16.17 ± 26.90	150.57–0.07	10.66 ± 10.74	55.00–0.27	0.857	0/0
IFN-γ	40.07 ± 30.59	230.87–3.36	46.01 ± 23.25	115.14–14.32	0.040*^3^	0/0
TNF-α	40.23 ± 27.98	232.33–8.35	44.74 ± 15.19	97.54–20.04	0.021*^3^	0/0
IP-10	377.59 ± 256.98	1712.98–116.17	501.43 ± 316.08	1781.90–143.82	0.002*^3^	0/0
MCP-1	―	―	―	―	―	―
MIP-1α	2.66 ± 1.65	13.48–0.71	2.99 ± 1.08	9.09–1.41	0.007*^3^	0/0
MIP-1β	27.14 ± 10.56	60.13–9.19	35.14 ± 19.50	138.81–12.35	0.002*^3^	0/0
PDGF-BB	128.85 ± 157.64	736.91–0.69	242.81 ± 245.57	1196.18–9.50	< 0.001*^3^	0/0
RANTES	2335.75 ± 1132.53	7825.46–84.60	2850.85 ± 1215.08	5794.57–181.78	0.031*^3^	0/0
VEGF	7.18 ± 7.74	31.57–0.11	6.68 ± 8.58	44.10–0.19	0.624	7/10

*^1^ The multiple linear regression analysis was conducted after adjustment for gender, age, and hospital.

*^2^The total number of plasma cytokine levels which were less than the limit of detection (LOD). The LOD was 0.02 pg/mL for IL-6, 0.13 pg/mL for IL-10, 0.18 pg/mL for IL-17A, 0.89 pg/mL for FGF-2, and 0.10 pg/mL for VEGF.

*^3^Significant at p < 0.05.

**Fig 1:**
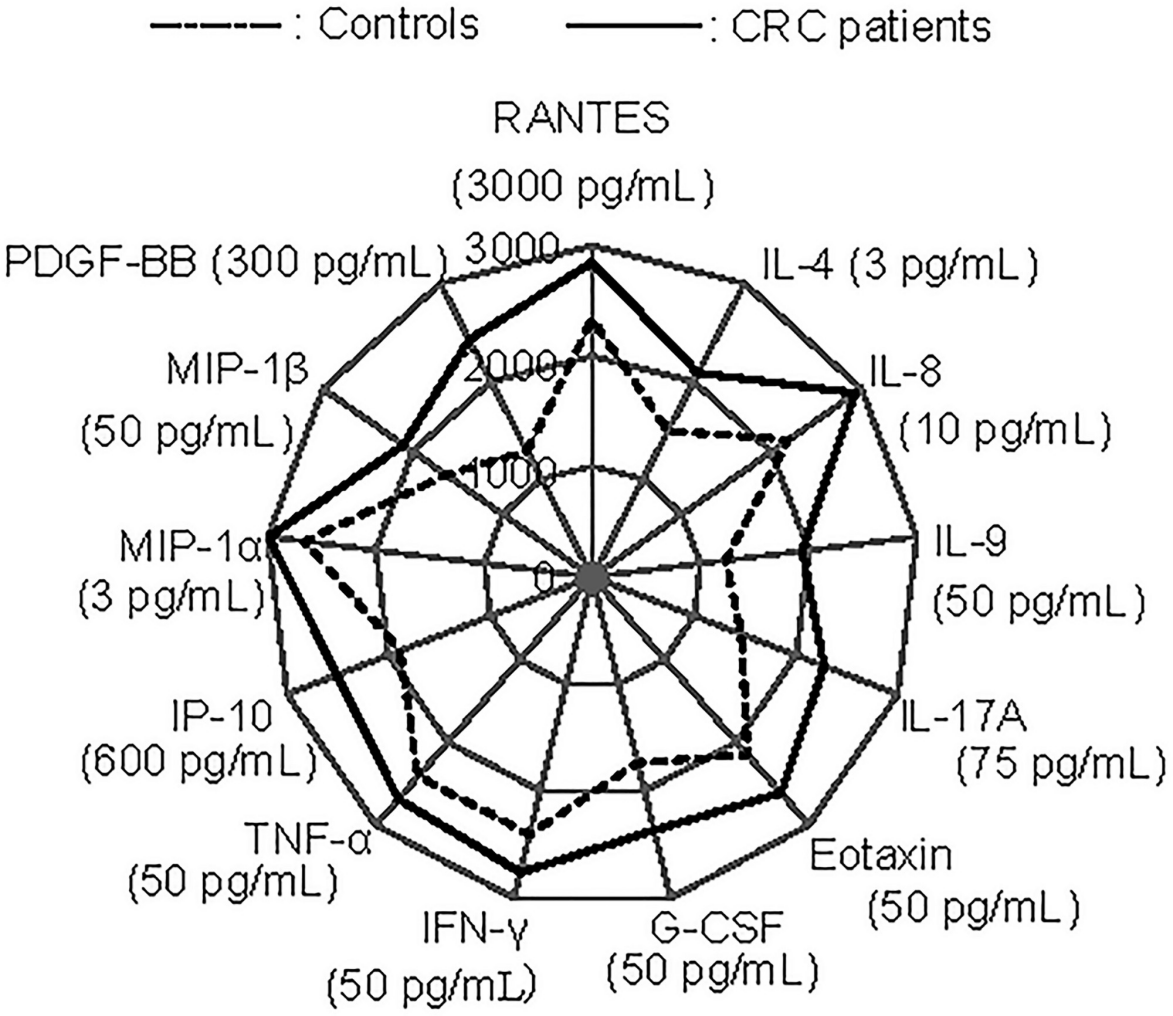
Comparison of plasma cytokine levels between CRC patients and controls, significant differences were observed by multiple linear regression analysis.

Significant differences were observed in the mean plasma levels between the CRC patients and controls for 13 plasma cytokines, namely IL-4, IL-8, IL-9, IL-17A, Eotaxin, G-CSF, IFN-γ, TNF-α, IP-10, MIP-1α, MIP-1β, PDGF-BB, and RANTES (p < 0.05, Fig 1). An additional comparison was conducted among the controls, stage 0 –II cases, and stage III–IV cases, and all 13 plasma cytokines, except IFN-γ and RANTES, showed an increasing trend according to tumour progression (S1 Table and S1 Fig). The associations of the above 13 plasma cytokines with the presence of CRC were all statistically significant (Table 3).

**Table 3:** Results of the logistic regression analysis of cytokines between CRC patients and controls after adjustment for gender, age, and hospital.

Cytokine	Regression coefficient	Standard error	p-value	Odds ratio (OR)	95% confidence interval (95% CI)		
IL-4	1.121	0.246	< 0.00001	3.068	1.895	-	4.967
IL-8	0.529	0.193	0.006	1.697	1.163	-	2.476
IL-9	1.187	0.251	< 0.00001	3.277	2.002	-	5.364
IL-17A	0.605	0.237	0.011	1.831	1.151	-	2.914
Eotaxin	0.695	0.213	0.001	2.003	1.320	-	3.039
G-CSF	0.806	0.215	0.0002	2.240	1.468	-	3.417
IFN-γ	0.382	0.184	0.038	1.465	1.022	-	2.099
TNF-α	0.442	0.188	0.019	1.556	1.077	-	2.250
IP-10	0.569	0.184	0.002	1.767	1.233	-	2.532
MIP-1α	0.506	0.189	0.008	1.659	1.144	-	2.404
MIP-1β	0.557	0.185	0.003	1.746	1.215	-	2.507
PDGF-BB	0.831	0.231	0.0003	2.295	1.460	-	3.607
RANTES	0.428	0.204	0.036	1.533	1.028	-	2.288

### Construction of a prediction model for the presence of CRC

As seen in Table 3, IL-9 and IL-4 showed the smallest p-values (< 0.00001) and were selected as the initial candidates for the IL-9 and IL-4 models, respectively. Seven plasma cytokines were nominated as additional candidates for each model: Eotaxin, G-CSF, TNF-α, IP-10, MIP-1α, MIP-1β, and PDGF-BB for the IL-9 model; and IL-8, Eotaxin, TNF-α, IP-10, MIP-1α, MIP-1β, and PDGF-BB for the IL-4 model. Finally, the backward elimination method revealed two multivariable prediction models (logistic models) for the presence of CRC as follows (p < 0.05, Table 4):

$$\begin{array}{l} IL‐9\;model\;:\;z\\ \;=1.643\;IL‐9+0.743\;Eotaxin+0.842\;G‐CSF-1.416\;TNF‐\alpha \\ \;-0.099\;gender-0.014\;age+0.989\;hospital\;B\\ \;+0.145\;hospital\;C+0.071\;hospital\;D \end{array}$$ (1)

$$\begin{array}{l} IL‐4\;model\;:\;z\\ \;=2.188\;IL‐4-0.642\;IL‐8+0.759\;Eotaxin+0.634\;IP‐10\\ \;-1.122\;TNF‐\alpha -0.138\;gender-0.013\;age\\ \;+0.917\;hospital\;B+0.456\;hospital\;C+0.275\;hospital\;D \end{array}$$ (2)

where hospitals B, C, and D are Saku General Hospital, Shinonoi General Hospital, and Hokushin General Hospital, respectively, and comparisons are with Matsushiro General Hospital.

**Table 4:** Calculated outcomes of the logistic regression analysis after adjustment for gender, age, and hospital.

Case	Cytokine	Regression coefficient	Standard error	p-value	Odds ratio (OR)	95% confidence interval (95% CI)		
IL-9 model	IL-9	1.643	0.377	< 0.001	5.170	2.468	-	10.832
	Eotaxin	0.743	0.272	0.006	2.102	1.232	-	3.585
	G-CSF	0.842	0.320	0.008	2.321	1.240	-	4.343
	TNF-α	-1.416	0.393	< 0.001	0.243	0.112	-	0.524
IL-4 model	IL-4	2.188	0.454	< 0.001	8.918	3.663	-	21.713
	IL-8	-0.642	0.320	0.045	0.526	0.281	-	0.986
	Eotaxin	0.759	0.277	0.006	2.137	1.242	-	3.678
	IP-10	0.634	0.232	0.006	1.884	1.195	-	2.971
	TNF-α	-1.122	0.358	0.002	0.326	0.161	-	0.657

### ROC analysis of a prediction model for the presence of CRC

**Fig 2:**
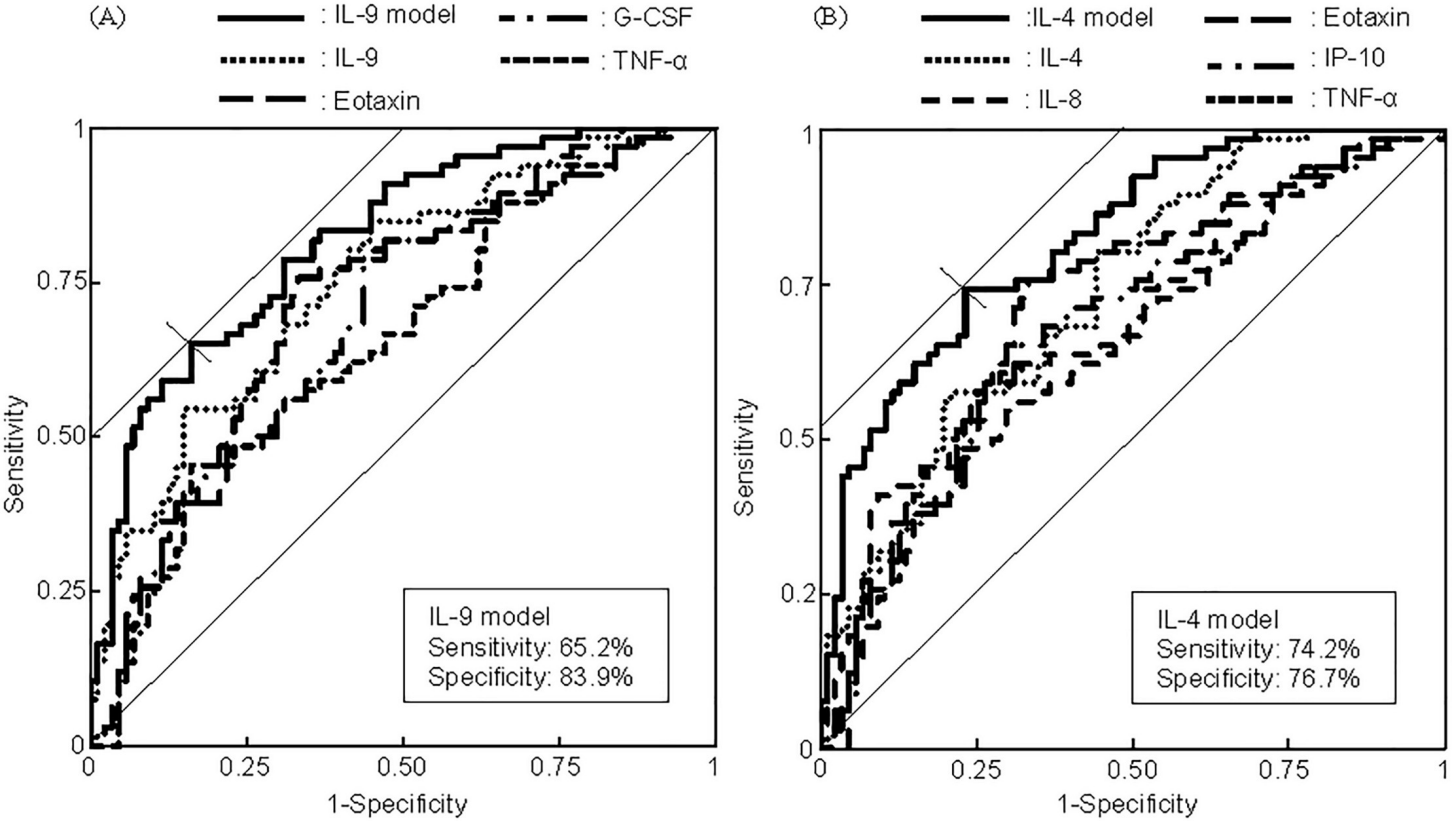
ROC analysis of the presence of CRC using a panel of select plasma cytokines that were most associated with CRC. (A) IL-9 model and (B) IL-4 model.

We performed ROC analysis to closely evaluate the above two prediction models for the presence of CRC (Fig 2). In the IL-9 model, the AUC, sensitivity, and specificity of the optimal cut-off points were 0.819, 0.652, and 0.839, respectively, while the corresponding values in the IL-4 model were 0.832, 0.742, and 0.767, respectively. Both models thus demonstrate “good” capability for discriminating between CRC patients and controls. A comparative prediction model without cytokines (model 0) was constructed by using gender, age, and hospital as variables. Its AUC, sensitivity, and specificity of the optimal cut-off points were 0.559, 0.485, and 0.655, respectively, therefore, the model demonstrated “poor” capability. All of the evaluation parameters for the IL-9 and IL-4 models were better than those for model 0. Also, single-cytokine models presented lower AUCs (0.657–0.755) than the multi-cytokine IL-9 and IL-4 models (Table 5).

**Table 5:** Results of ROC analysis of two multivariable prediction models and each cytokine.

Case	Sensitivity	Specificity	Area Under the ROC Curve AUC
Model IL-9	0.652	0.839	0.819
IL-9 Eotaxin G-CSF TNF-α	0.848	0.552	0.755
Eotaxin	0.758	0.667	0.714
G-CSF	0.788	0.563	0.695
TNF-α	0.455	0.839	0.657
Model IL-4	0.742	0.767	0.832
IL-4	0.576	0.791	0.736
IL-8	0.591	0.736	0.680
Eotaxin	0.758	0.667	0.714
IP-10	0.652	0.678	0.681
TNF-α	0.455	0.839	0.657

## Discussion

The study results supported our working hypothesis that the levels of some plasma cytokines vary depending on the presence of CRC. Even after controlling for gender, age, and hospital, the plasma levels of CRC patients and controls differed significantly in terms of the following 13 cytokines: IL-4, IL-8, IL-9, IL-17A, Eotaxin, G-CSF, IFN-γ, TNF-α, IP-10, MIP-1α, MIP-1β, PDGF-BB, and RANTES (Table 2 and Fig 1, p < 0.05). The combinatorial assessment of some of these plasma cytokines showed promise for detecting the presence of CRC. In fact, the ROC analysis showed that the IL-9 and IL-4 models had “good” capability for discriminating between CRC patients and controls (Table 4 and Fig 2). These two models showed similar AUC values, although some differences were observed; the performance of the IL-9 model was excellent with respect to specificity, while the IL-4 model balanced both sensitivity and specificity (Table 5).

In our research, the logistic regression analysis revealed that the cytokines IL-9 and IL-4 had potential association with the presence of CRC (Table 3). IL-9 is a cytokine produced by CD4^+^ Th2 cells as well as by some B lymphomas; it has been shown to induce an increase in the proliferation of CRC cells and to promote tumorigenesis in CRC cells [24]. Kantola et al. conducted a screening cohort study using cytokines in serum, including IL-9, and concluded that cytokine biomarkers might be a promising tool for the detection of CRC [25]. Broadly, IL-4 can be categorized as a type of anti-inflammatory cytokine. IL-4 and IFN-γ are the most frequently described cytokines in the inflammatory process. Szylberg et al. analyzed 144 colorectal polyps and showed a significantly increased level of IL-4 in adenomas, serrated adenomas, and hyperplastic polyps compared with the control group [26]. Sharp et al. reported that levels of the anti-inflammatory cytokine IL-4 were significantly elevated in advanced CRC, whereas IFN-γ levels were not statistically different [27]. Recent studies have found that IL-4 levels in colorectal polyp-derived serum were significantly higher than those in serum from healthy volunteers [28].

Other cytokines included in the IL-9 and IL-4 models warrant mention. There is evidence that the cytokine Eotaxin is strongly associated with primary and metastatic tumors of colorectal origin [29]. Both IL-4 and IL-13 synergistically enhance TNF-α–induced Eotaxin production [30]. Natori et al. suggested that G-CSF may have the potential to promote tumor growth, at least in part, by stimulating angiogenesis [31]. The level of G-CSF has been examined in CRC patients and found to be significantly higher than in healthy subjects [32]. Despite the small sample size, another study found significantly higher levels of G-CSF in CRC patients before surgery compared with controls at baseline [33]. TNF-α is a potent pro-inflammatory cytokine thought to be involved in the pathogenesis of inflammatory bowel disease [34] and has been reported to promote inflammation and colitis-associated cancer [35]. In the blood of the patients with CRC, a significant elevation has been reported in the levels of TNF-α [36,37]. Dimberg et al. analyzed 50 CRC patients and found a significantly higher IL-8 (CXCL 8) level in cancer tissue compared with paired normal tissue, and showed that CRC patients exhibited significantly higher plasma levels than healthy controls [38]. Crucitti et al. conducted screening of 30 CRC patients; although the sample size was small, significantly higher levels of IL-1β, IL-7, IL-8, G-CSF, IFN-γ, and TNF-α were detected in CRC patients compared with controls at baseline [39]. The CXCL10 (IP-10)/CXCR3 axis of inflammatory mediators is one of the most important chemokine axes and has been proven to be a lymphocyte‑associated metastasis mediator in several tumors [40], although in one, report serum levels of IP-10 were of measureable concentration but did not differ between the control and CRC groups [41].

Several studies have investigated validity of the Bio-Plex multiplex bead array immunoassay system (originally developed by Luminex Co.) by comparison with traditional enzyme-linked immunosorbent assay (ELISA) [42–45]. Carson et al. conducted a simultaneous quantitation of 15 cytokines using the multiplex immunoassay system in parallel with quantitation by ELISAs, and showed that use of the multiplex bead array did not reduce sensitivity for any cytokines including IL-1β, IL-2, IL-4, IL-5, IL-6, IL-9, IL-10, IL-13, GM-CSF, IFN-γ, TNF-α, and MCP-1 compared with that of the ELISAs [42]. Prabhakar et al. indicated that overall values of the cytokines IL-1β, IL-6, IL-8, and TNF-α were not significantly different between the two types of assays [43]. We performed a similar validation study by measuring IL-9, and calculated a Spearman’s correlation coefficient of 0.47 between the Bio-Plex multiplex bead array and ELISA (unpublished data). Compared with ELISA, the Bio-Plex system showed a more appropriate range of circulating IL-9 concentrations, which is in line with the previous reports [46–47]. Although the multiplex bead array immunoassay has emerged as a suitable option for measuring several plasma cytokines at once, extrapolation of our results should be made with caution, particularly with regard to plasma cytokine data obtained by other assays.

In our study, the levels of several plasma cytokines varied significantly between CRC patients and control subjects, suggesting the possibility of the differential expression of plasma cytokines as potential biomarkers for detecting the presence of CRC. Our results should be validated in other populations and should be supported by future studies to examine the temporal changes in plasma cytokine levels according to colorectal cancer progression and prognosis. Lengthy and careful consideration should be given why IL-9 and IL-4 strongly correlated with the presence of CRC.

## Supporting information

S1 Table Results of multi-analysis of cytokines; comparisons between stage 0 –II patients (42 cases; 23 males and 19 females), stage III–IV patients (24 cases; 13 males and 11 females), and controls by multiple linear regression analysis. Significant differences were observed in mean plasma levels for IL-4, IL-8, IL-9, IL-17A, Eotaxin, G-CSF, TNF-α, IP-10, MIP-1α, MIP-1β, and PDGF-BB between CRC patients and controls (p < 0.05).
(TIF)

S1 Fig Comparison of plasma cytokine levels between stage 0 –II patients (42 cases; 23 males and 19 females), stage III–IV patients (24 cases; 13 males and 11 females), and controls. Significant differences were observed for IL-4, IL-8, IL-9, IL-17A, Eotaxin, G-CSF, TNF-α, IP-10, MIP-1α, MIP-1β, and PDGF-BB (p < 0.05) using multiple linear regression analysis.
(TIF)
